# Hand Sanitizer Gels: Classification, Challenges, and the Future of Multipurpose Hand Hygiene Products

**DOI:** 10.3390/toxics11080687

**Published:** 2023-08-10

**Authors:** Yilei Ma, Jia Yi, Jiahui Ma, Haiyang Yu, Li Luo, Wei Wu, Libo Jin, Qinsi Yang, Ting Lou, Da Sun, Min Cao

**Affiliations:** 1 Institute of Life Sciences & Biomedical Collaborative Innovation Center of Zhejiang Province, Wenzhou University, Wenzhou 325035, China; 2 Affiliated Dongguan Hospital, Southern Medical University, Dongguan 523059, China; 3 Bioengineering College of Chongqing University, Chongqing 400044, China; 4 Wenzhou Institute, University of Chinese Academy of Sciences, Wenzhou 325000, China; 5 Yiwu Center for Disease Control and Prevention, Yiwu 322000, China; louting22@163.com; 6 The Quzhou Affiliated Hospital of Wenzhou Medical University, Quzhou People’s Hospital, Quzhou 324000, China

**Keywords:** hand sanitizer gels, antimicrobial properties, biological functions, hand hygiene

## Abstract

Hand hygiene is a crucial measure in the prevention and control of infections, and there is a growing awareness among individuals who are making a conscious effort to maintain hand cleanliness. With the advent of the SARS-CoV-2 outbreak, the demand for hand hygiene products has also gradually shifted towards those with antimicrobial properties. Among these products, hand sanitizer gels (HSGs) have gained considerable popularity as an efficient method of hand cleaning, due to their rapid drying and sustained antimicrobial efficacy. Concurrently, there has been a growing interest in novel HSGs that offer additional functions such as skin whitening, moisturizing, and anti-inflammatory effects. These novel HSGs effectively address concerns associated with the ingestion of antimicrobial ingredients and demonstrate reduced skin irritation, thereby alleviating hand dermatological issues. This review provides an extensive overview of the application scenarios, classification, and challenges associated with HSGs while emphasizing the emergence of novel components with biological functions, aiming to contribute to the advancement of hand hygiene practices and offer novel insights for the development of novel HSGs with outstanding antimicrobial properties with other multiple biological functions and desirable biosafety profiles.

## 1. Introduction

Hand hygiene plays a crucial role in preventing infections and controlling the spread of diseases, as hands serve as a major medium for the transmission of pathogenic microorganisms [1,2]. Notably, over half of respiratory viruses and enterovirus infections, along with bacterial infections, are transmitted through hand-to-mouth contact among people living in close contact [3,4,5]. Therefore, hand hygiene is crucial in preventing infections and controlling the spread of epidemics, which can ultimately safeguard human life and health [6,7]. In response to the global COVID-19 pandemic, the demand for hand cleaning products with antimicrobial properties has surged [8,9]. Instant hand sanitizers (IHSs) have gained significant popularity due to their effectiveness in reducing hand pathogens, along with their convenience, accessibility, and wide consumer acceptance [10,11]. Compared to traditional soaps and wipes, IHSs are considered more convenient and efficient in reducing bacterial counts [12]. Consequently, using IHSs as an alternative to disposable wipes before meals is considered more convenient and efficient [13]. As the importance of hand hygiene continues to be emphasized, promoting the use of IHSs as an integral part of everyday routines can significantly contribute to reducing the transmission of infectious diseases and improving overall public health.

IHSs typically contain active functional ingredients, as well as inactive carrier ingredients, such as excipients, humectants, fragrances, and colorants [14,15]. They are available in various forms, including gels, liquids, sprays, and foams [16]. Each formulation has its own characteristics and considerations: (I) The low viscosity of the liquid form makes it more challenging to use effectively [17]; (II) The spray formulation of IHSs offers broader coverage, allowing for convenient application to larger surfaces. However, it requires a valve mechanism and poses a safety risk due to the flammability of ethanol. Extra precautions must be taken to avoid accidents and ensure proper storage [18]; (III) The high humidity of the foam formulation can reduce its antimicrobial properties and prolong the drying time, leading to discomfort [19]. In comparison, hand sanitizer gels (HSGs) are a popular choice due to their rapid drying time and continuous antimicrobial efficacy [20]. Additionally, HSGs form a protective layer at the application site, providing a longer period of protection on the skin. It is essential to rub HSGs thoroughly to ensure complete coverage of the hand skin (see Table 1) [21,22]. It is important to consider factors such as ease of use, safety, antimicrobial efficacy, and user comfort when selecting the most suitable IHSs for personal hygiene practices. This review will take HSGs as the main character to introduce their research progress in the field of hand hygiene.

Since the onset of the pandemic, many countries have emphasized the implementation of non-pharmaceutical preventive measures, prominently including the intensive use of HSGs [24]. Additionally, the critical role of hand sanitizers in reducing the transmission of infectious diseases such as COVID-19 has led to a surge in global demand for HSGs since 2020, resulting in a remarkable 600-fold increase in market production [25,26]. Consequently, there has been a significant decrease in the incidence of infectious diseases in recent times [27]. This surge in market sales of HSGs has spurred the refinement of their applications, resulting in the development of a wide range of novel products with specific biological functions. These products have been designed to meet the varying needs of different consumer groups.

Herein, we focus on the research progress of HSGs in hand hygiene, highlighting the application scenarios, challenges, and possible solutions, as well as the outlook for the future application of biological functions. By making informed decisions, individuals can enhance their hand hygiene routine and contribute to the prevention of infections and the control of disease transmission.

## 2. Application Scenarios for HSGs

**Figure 1:**
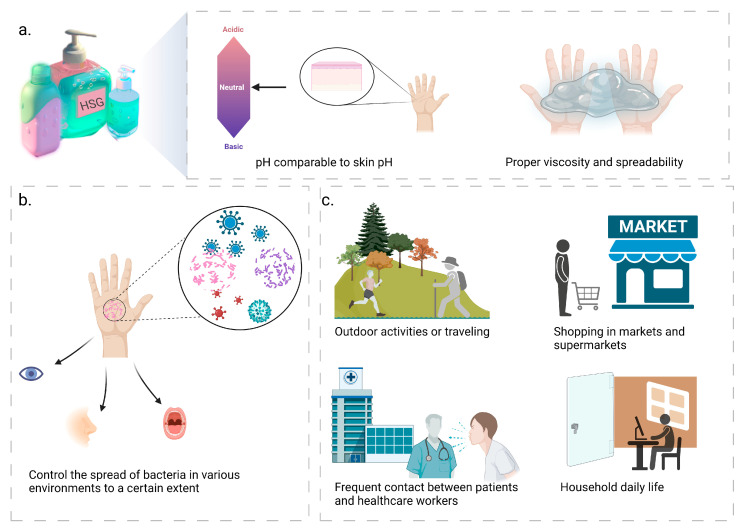
Hand sanitizer gels (HSGs) possess favorable sensory characteristics, exert a certain degree of control over bacterial transmission, and can be applied in various scenarios. (**a**) Hand hygiene products with good sensory properties, including pH similar to skin, as well as appropriate viscosity and spreadability; (**b**) HSGs can control the spread of bacteria in various environments to a certain extent and significantly reduce the transfer of microorganisms, thus effectively reducing respiratory and gastrointestinal infections; (**c**) The versatility of HSGs extends to multiple scenarios, embracing outdoor activities, supermarket shopping, health centers, domestic routines and other scenarios.

Hand hygiene products that are widely accepted for use should have good sensory characteristics, which include a pH value similar to that of the skin, as well as appropriate viscosity and spreadability [22], as depicted in Figure 1a. Additionally, it is important not to develop an allergic reaction to the skin. The majority of hand hygiene products contain high levels of alcohol, which can cause skin irritation and dryness, as well as harm the environment [28]. Therefore, it is essential to find hand hygiene products that are safe and environmentally friendly. Products with poor sensory characteristics can reduce the frequency with which it is necessary to wash your hands [29]. The components of HSGs are biocompatible with each other at an excellent level, and the most abundant component is the thickening agent, mainly using carbomer, which is extensively used for skin and eye wound healing [30,31]. Simultaneously, carbomer has high viscosity at low concentrations and has the advantages of a wide viscosity range, great flowability, compatibility with many active ingredients, high transparency, good thermal stability, and high consumer acceptance [32,33].

HSGs can be utilized in a wide variety of situations, owing to its effectiveness in minimizing respiratory and gastrointestinal infections based on its ability to control to a certain extent the spread of bacteria in a range of environments and to significantly reduce the transfer of microorganisms [34,35,36], as illustrated in Figure 1b,c. (I) When engaging in outdoor activities or traveling, the compact size of the HSGs allows for convenient and hygienic hand cleaning without the necessity of additional water sources and towels, thus avoiding infections and contagious diseases caused by the presence of pollutants and bacteria [37], making HSGs suitable for travel, camping, and other activities [10]; (II) during shopping in markets and supermarkets, customers frequently touch surfaces such as cart handles and shelves, resulting in bacterial contamination and transmission through hand-to-mouth and other hand-to-face touching behaviors [37,38]. In pursuit of creating a safe, healthy, and hygienic environment, HSGs offer a convenient alternative to hand soaps and other cleaning methods. They effectively eliminate hand bacteria, ensuring personal cleanliness [39]; (III) frequent contact between patients and healthcare workers can lead to the spread of bacteria in health centers such as hospitals, clinics, and pharmacies, where HSGs are more effective, act faster and can be made available at the point of patient care [40]. Studies have also shown that HSGs are generally better tolerated by the skin than soap and water [41]; (IV) family members use mobile phones, keyboards and other electronic devices daily. It is possible to spread bacteria to the hands by touching these devices [42]. In addition to furniture, doorknobs, and other common items, family members can also spread bacteria from one item to another. Therefore, using HSGs is a quick, efficient, and convenient way to help maintain good hand hygiene and prevent the spread of bacteria [34,43].

## 3. Classification of HSGs

HSGs can be classified into two categories based on their active ingredients: Alcohol-based HSGs (ABHSGs) and Non-alcohol-based HSGs (NABHSGs). Both types are effective in inhibiting microorganisms on the hands and no significant difference in efficacy has been observed between them [44]. Nevertheless, the mechanisms of action for these two HSGs types differ [14].

### 3.1. ABHSGs

ABHSGs exert rapid antimicrobial effects by denaturing and deactivating microorganisms on the hands through their interaction with membrane proteins, thus acting as effective antimicrobial agents [1]. However, the alcohol-based components present in ABHSGs can often adversely affect the lipid structure of epidermal keratinocytes, resulting in side effects such as hand dryness and cracking [45].

Typically, commercially available ABHSGs contain primary ingredients such as ethanol (ranging from 60% to 95% concentration), isopropanol, or a combination of both [29,46]. Additional ingredients like hydrogen peroxide, water, and notably, glycerol as emollients, can be incorporated into the product to reduce irritation and minimize the occurrence of contact dermatitis [47]. ABHSGs have been demonstrated to eliminate most microorganisms present on the hands, and the alcohol group within the product acts as a potent antimicrobial agent against a broad spectrum of bacteria, viruses, and fungi, including methicillin-resistant *Staphylococcus aureus*, *E. coli*, H1N1, and the Ebola virus [48,49,50]. The World Health Organization has confirmed that corresponding concentrations of these alcohols effectively inactivate SARS-CoV-2 [51]. Ethanol is particularly effective against non-enveloped viruses, making it the primary active ingredient in most HSG products [52,53]. The antimicrobial activity of ABHSGs largely depends on the percentage concentration of alcohol-based substances [54]; if the content falls below 60% *v*/*v*, product quality may be compromised [16]. In response to the COVID-19 pandemic, the FDA has recommended increasing the concentration of isopropanol to 91% *v/v* and ensuring that the ethanol concentration does not exceed 94.9% *v/v* [46].

ABHSGs typically achieve their full antimicrobial activity within 15 s [55]. In accordance with EN1500 guidelines, approximately 3 mL of ABHSGs should be applied to the hands for approximately 30 s to ensure their effectiveness against microorganisms [29,56]. Nonetheless, factors such as the amount of ABHSGs applied, the duration of application, and hand size can influence the reduction of microbial load, the extent of hand surface coverage, and the drying time [57,58]. A significant correlation has been observed between hand area and the number of detected CFUs [59]. Larger hand surfaces necessitate a greater amount of ABHSGs to guarantee complete coverage, which, in turn, prolongs the required duration of hand friction. This could decrease the acceptability, practicality, and ultimately, the sterilization efficacy of ABHSGs [60].

### 3.2. NABHSGs

The efficacy of NABHSGs in reducing the incidence and transmission of bacterial infections has been demonstrated [61]. The antimicrobial function of NABHSGs is primarily mediated through the presence of cationic surfactants, such as quaternary ammonium compounds (QACs) [62]. These compounds exhibit a broad spectrum of antimicrobial properties due to their hydrophilic and hydrophobic components, specifically the positively charged ammonium cations in their hydrophilic part [37,63]. The presence of both hydrophilic and hydrophobic components enhances their surface activity and the ability to form liposomes, thereby increasing their antibacterial capacity [64,65]. Furthermore, ingredients such as aloe vera, glycerol, and vitamin E can be incorporated into NABHSGs to reduce skin irritation and enhance their moisturizing effects [22].

Benzalkonium chloride (BAK), a representative of QACs, exhibits excellent sterilization performance within 30 s and has been widely employed in medical institutions and cleaning supplies as a non-alcohol-based functional antimicrobial agent for NABHSGs [66,67]. Compared to alcohol, QACs offer several advantages for hand disinfection: (1) they are non-toxic, less irritating to the skin, and non-flammable [68]; (2) the combination of ABHSGs and NABHSGs (primarily referring to benzalkonium chloride) generates a synergistic effect that enhances the antimicrobial range and efficacy of the alcohol group while also providing an immediate and long-lasting antimicrobial effect [69]. Therefore, the identification of various QACs components with antimicrobial activity and good biocompatibility could potentially address future challenges associated with alcohol demand and side effects.

## 4. Application Challenges of HSGs

### 4.1. Antimicrobial Functional Ingredients

**Figure 2:**
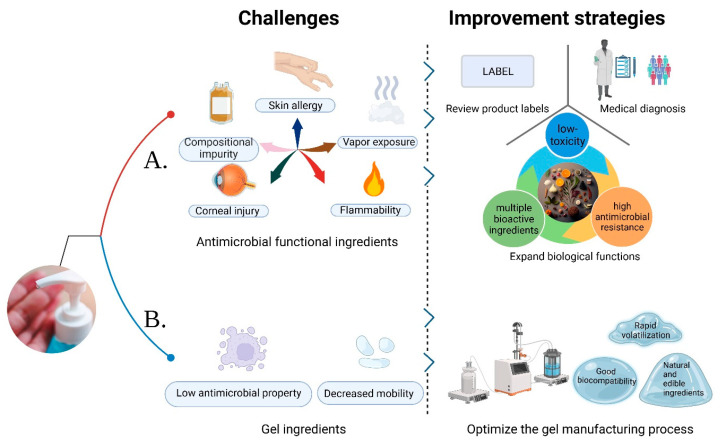
Application challenges and the corresponding improvement strategies of hand sanitizer gels (HSGs). (**A**) The challenges of antimicrobial functional ingredients at present and the corresponding improvement measures are put forward; (**B**) The current challenges associated with gel ingredients are identified, along with proposed measures for their improvement.

Since the onset of the COVID-19 pandemic, there has been a significant surge in sales of ABHSGs [70]. To meet the challenge of increased demand and raw material shortages, many countries have permitted manufacturers and medical institutions to develop and produce hand sanitizers independently. Unfortunately, this has led to a situation where some manufacturers fail to adhere to proper quality control measures or use denatured alcohol, resulting in a notable increase in substandard hand sanitizer products in the market [47,71,72,73]. Furthermore, the fermentation and distillation processes employed by manufacturers, along with the impact of production equipment and the environment, may introduce impurities such as benzene and acetaldehyde into hand sanitizers, exacerbating public health concerns [74,75]. Consequently, it is recommended that users critically evaluate the antimicrobial functional ingredients in hand sanitizer formulas and carefully review product labels to avoid potential allergens based on their personal sensitivities before use [76], as shown in Figure 2.

Common antimicrobial components found in regular hand sanitizer products (such as ABHSGs) can cause corneal epithelial cell destruction at high concentrations, leading to eye diseases. Prolonged contact with hands may also increase the risk of local adverse reactions, such as contact dermatitis and atopic dermatitis [77]. In recent years, clinical cases involving adverse effects from ABHSGs have been on the rise, particularly among children [78,79]. Children tend to use hand sanitizers more frequently than adults [80], and face an increased risk of ingestion and exposure problems after using informal ABHSGs, underscoring the importance of adult supervision during use [78,81]. Additionally, healthcare workers who apply ABHSGs before handling newborns may heighten the risk of neonatal exposure to ethanol vapor, potentially causing neuronal damage and leading to neurodevelopmental delay and behavioral issues. However, the impact of such low-dose ethanol exposure on neonatal brain development is currently unclear [82,83]. Furthermore, isopropanol ingestion can result in common health problems. Accidental inhalation or dermal exposure may cause poisoning, although it usually does not lead to serious health hazards [84]. Repeated exposure of microbes to disinfectants, antibiotics, and other genotoxic chemicals leads to the development of resistance, becoming a significant global concern, particularly burdening healthcare professionals [85]. Triclosan serves as a prime example, having been used as an antimicrobial component in NABHSGs over the past decades, but evidence indicates its potential for environmental impact and antibiotic resistance [86]. Therefore, excessive and prolonged use of HSGs also entails potential risks. Furthermore, the impact of ethanol, isopropanol, and other ingredients on the environment has long been a topic of concern [87]. These compounds can volatilize or seep into soil and groundwater, significantly affecting aquatic organisms [88]. Thus, while addressing potential issues associated with the long-term use of HSGs, it is crucial to emphasize effective management of good hygiene practices.

To maintain hand hygiene, particularly among children and healthcare workers, frequent hand washing is crucial. Therefore, it is important to select an emollient that effectively strengthens the skin barrier. Research indicates that BAK, an antiseptic ingredient, provides immediate and long-term antimicrobial effects, and the addition of emollients typically does not diminish its effectiveness [89]. Furthermore, BAK tends to be less irritating to the skin and rarely causes allergic reactions [63]. In cases where adverse reactions, such as hand rashes, arise from using ABHSGs, it is recommended to either switch to hand sanitizers containing BAK or seek medical treatment [90]. It is important to note that ethanol and isopropanol, commonly found in ABHSGs, are volatile and combustible substances that could cause fires when used near flames or exposed to high temperatures [16,18,91]. As such, the inclusion of novel safe antimicrobial ingredients can offer a safer alternative with an improved safety profile.

### 4.2. Gel Ingredients

The stability of the gel is related to the pH of the compound, with a lower pH resulting in decreased mobility. Considering that ethanol and isopropanol have different polarities, and isopropanol exhibits a significantly lower polarity compared to ethanol, a greater amount of triethanolamine must be incorporated into isopropanol to produce a stable polymer with carbomer [92]. Studies have demonstrated that anionic thickeners (such as carbomer or acrylate) notably impair the antimicrobial persistence of ABHSGs on the skin. As an alternative, non-ionic polymer thickeners like hydroxypropyl cellulose may be considered to replace carbomer and improve antimicrobial persistence [93]. In addition, by optimizing the manufacturing process of the gel, it is possible to explore materials with good biocompatibility, fast volatilization, and even completely natural edible ingredients in HSGs, which not only contribute to the stability of the gel, but also provide enhanced biocompatibility, leading to faster volatilization. This exploration opens up the possibility of reducing reliance on synthetic ingredients and instead adopting entirely natural and edible ingredients. The trend towards using natural and edible ingredients in hand hygiene products is in line with consumers’ growing preference for environmentally friendly and safe options.

## 5. Novel Antimicrobial Ingredients of HSGs

**Figure 3:**
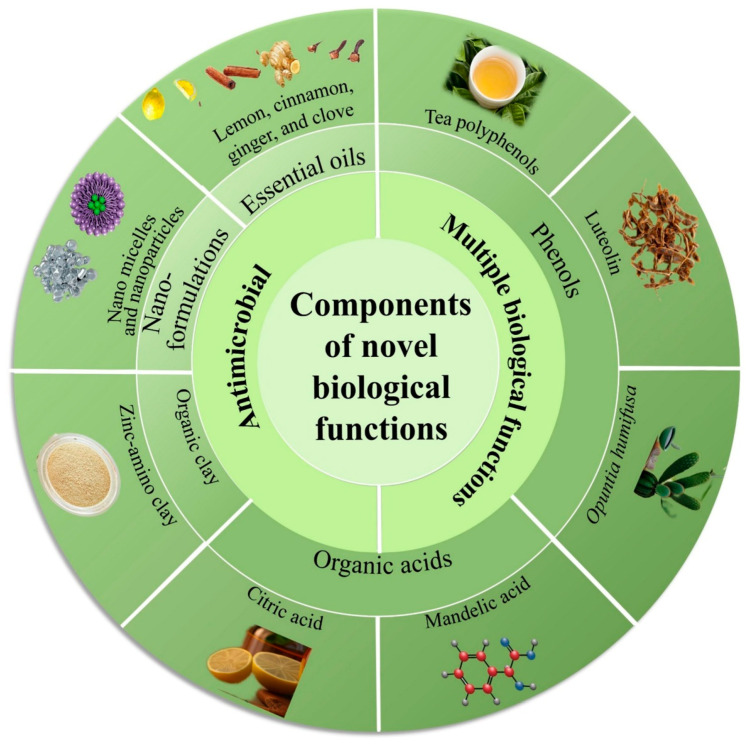
Novel components of hand sanitizer gels (HSGs) possess antimicrobial properties and exhibit multiple biological functions.

**Figure 4:**
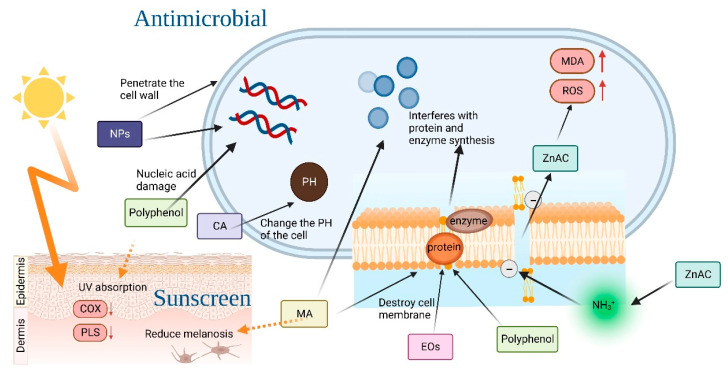
The antimicrobial mechanisms of essential oils, phenols, and organic acids, as well as the skin whitening and anti-inflammatory mechanisms of phenols and Mandelic acid (MA).

Due to various biocompatibility issues associated with antimicrobial components in traditional HSGs, there has been a notable shift towards the use of novel HSGs [88,90], as illustrated in Figure 3. Novel HSGs are embracing the use of natural and safe antimicrobial substances, such as essential oils, phenols, organic acids, and others, aiming to reduce or even completely replace traditional ingredients like ethanol or isopropanol. Additionally, NABHSGs represented by cationic surfactants have gained attention as an alternative [94]. The research community and consumers have also exhibited a growing interest in antimicrobial agents such as organic clay and nanoformulations. These agents not only provide antimicrobial effects but also offer certain anti-inflammatory and whitening properties, as shown in Figure 4. This dual functionality has contributed to their increasing popularity among researchers and consumers alike [95,96].

### 5.1. Essential Oils

Natural substances with antimicrobial activity have been extensively investigated and studied [97]. Among these, natural plant essential oils (EOs) have been found to affect the synthesis of bacterial lipid membranes, including certain proteins and enzymes on the membrane surface, due to their lipid-soluble nature. This makes the bacterial membranes more permeable and reduces the protein content of the bacterium, while increasing the hydrophobicity of the bacterial cell surface, affecting various cellular processes that ultimately lead to cell death [22]. Furthermore, EOs have a stronger impact on the cell wall of gram-positive bacteria than on the cell membrane [98]. By combining representative EOs, namely lemon, cinnamon, ginger, and clove, the novel NABHSGs can be developed with superior antimicrobial properties compared to 60% ethanol, as demonstrated by the synergistic action of the main components limonene, cinnamaldehyde, turmeric, and eugenol [99,100,101]. Additionally, M. Ismail et al. prepared a novel HSG with pepper essential oil, clove essential oil, and a small amount of ethanol. This novel HSG reduces the exposure risk of isopropanol and alcohol on the skin and exhibits good drug diffusion and antibacterial properties [102].

The utilization of natural substances, particularly essential oils, in the development of novel HSGs presents a promising approach to enhance their antimicrobial efficacy while minimizing potential risks associated with traditional antimicrobial components. By leveraging the synergistic effects and unique properties of these natural substances, researchers are able to explore novel avenues for the formulation of safer and more effective HSGs.

### 5.2. Phenols

Tea polyphenols have multiple biomedical functions, with active components like epigallocatechin gallate (EGCG) and epigallocatechin-3-gallate-palmitate (EC16) directly damaging the cell membrane, altering cell structure, and affecting the proteins and DNA of microorganisms to exhibit antimicrobial effects. In particular, EC16, the main component of tea polyphenol palmitate, has emerged as a novel safe and non-toxic HSG component [103,104]. Research by M. Xi et al. has found luteolin (LUT) to be a highly promising antimicrobial functional component of hand sanitizers. LUT increases cell membrane permeability to inhibit bacterial growth, causing significant and irreversible damage to bacterial structure and leading to K^+^ extravasation. Additionally, LUT has strong antioxidant and free radical scavenging abilities, protecting cells from damage. As a novel HSG component, LUT not only exhibits antimicrobial effects, but also absorbs both ultraviolet A (UVA) and ultraviolet B (UVB) radiation, reducing their adverse effects on the skin. LUT can effectively mitigate the generation of reactive oxygen species (ROS) in the skin induced by ultraviolet (UV) radiation through mechanisms such as stabilizing free radicals, inhibiting pro-oxidant enzymes, and inducing antioxidant enzymes. Furthermore, LUT demonstrates effective inhibition of pro-inflammatory signaling pathways induced by UV radiation by targeting pro-inflammatory cytokines like IL-6, or COX-2, or NF-κB-dependent pathways [105,106].

*Opuntia humifusa* extract (OHE) is high in total polyphenols and flavonoids, giving it antioxidant and antimicrobial activity, as well as high moisture retention capabilities [107]. OHE stimulates the expression of epidermal hyaluronic acid synthases (HAS) and regulates the synthesis of hyaluronic acid (HA), thereby reducing the expression of the hyaluronidase transcript and contributing to skin hydration [25]. Additionally, OHE has whitening and anti-wrinkle effects. A study by Park et al. [108] demonstrated that OHE could regulate the ability of the skin to produce HA induced by UVB. OHE also inhibited the compensatory increase of HA, hyaluronic acid-binding protein (HABP), and CD44 protein expression under UV irradiation, all while reducing HAS percutaneous water loss and erythema. This suggests that OHE may protect the skin from the harmful effects of UVB radiation.

### 5.3. Organic Acids

Organic acids (OAs) act as broad-spectrum antimicrobial substances, inhibiting the growth of microorganisms through various mechanisms, and show synergistic antimicrobial effects with alcohol. Citric acid (CA), for instance, can alter the pH value of cells, thereby interfering with the normal metabolism of bacteria and exerting antimicrobial effects [109,110]. Using organic acids, alone, as antimicrobial ingredients in hand sanitizers is also significant. Mandelic acid (MA) has been identified as an antimicrobial component of NABHSGs, playing an antimicrobial role by affecting the bacterial cell membrane and inhibiting bacterial protein synthesis. Results of experiments by Pavlína Egner et al. suggest that MA has stronger antimicrobial properties and better stability than alcohol groups [111]. In addition to its antimicrobial properties, MA can also be employed as an active ingredient to reduce pigmentation and prevent skin erythema, sunburn, and skin cancer caused by ultraviolet rays. Lower concentrations of MA promote the regeneration of cells in different layers of the skin, reducing damage to the skin barrier and dehydration of the stratum corneum, thereby leading to maintenance of optimal pH values and a revamp of photoaging [112].

By harnessing the potential of these organic acids, researchers can develop novel HSGs that provide improved antimicrobial activity while promoting skin health and overall well-being.

### 5.4. Organic Clay

Zinc-amino clay (ZnAC) is a type of organic clay characterized by its strong antimicrobial properties and minimal toxic side effects. In an aqueous solution, ZnAC-HSG can interact with negatively charged bacterial lipid membranes through electrostatic interactions involving the quaternary ammonium (-NH_3_^+^) groups of organically modified clay. This interaction disrupts the permeability of the phospholipid bilayer and cell membrane, leading to an increased uptake of ZnAC by bacterial cells. Subsequently, intracellular ROS concentration rises, malondialdehyde (MDA) production is significantly increased, and ultimately, cell death occurs due to bacterial membrane disruption [25].

Moreover, the presence of organic clay in the sunscreen enhances the viscosity of its oil phase, resulting in the formation of a uniformly distributed film that improves the sunscreen’s ability to absorb ultraviolet rays. Consequently, sunscreen products containing HSGs with organic clay components exhibit enhanced sun protection and whitening effects [113].

### 5.5. Nanoformulations

Nanoformulations have emerged as a promising approach for enhancing the stability and biocompatibility of antimicrobial agents, leading to their increasing adoption in novel NABHSGs. For instance: (Ⅰ) Rhamnolipid nano-micelles exhibit antimicrobial activity comparable to NABHSGs, while maintaining non-cytotoxic properties. The inclusion of (biological) surfactants enhances the hydrophobicity of degrading microorganisms, thereby rendering the hydrophobic matrix more accessible to cells [114]. In comparison to ABHSGs, Rhamnolipid nano-micelles are safer and exhibit minimal impact on the skin [115,116]. Not only do Rhamnolipid nano-micelles possess antimicrobial properties, but they also hold potential as a therapeutic agent for COVID-19 treatment in future research [117]; (Ⅱ) nanoparticles, measuring between 1 and 1000 nm in size, have been reported to exhibit antimicrobial activity against both viruses and bacteria [118]. Silver nanoparticles, in particular, are excellent materials in the biomedical field [119,120]. Synthesized from plant extracts, silver nanoparticles are not only safe in composition but also demonstrate potent antimicrobial effects [121]. Their high surface area-to-volume ratio enables them to penetrate bacterial cell walls, alter membrane structures, and even eliminate target cells. Silver nanoparticles exert their effects through the release of silver ions, increased cell membrane permeability, generation of reactive oxygen species, and interference with DNA replication [120]. For instance, moss and eucalyptus leaf extracts have been employed in the preparation of silver nanoparticles [122]. The eucalyptus leaf extract exhibited an inhibitory effect on bacterial biofilm formation and demonstrated more favorable antimicrobial effects [123,124]. Furthermore, chitosan serves as a coating on the surface of silver nanoparticles, acting as a stabilizer to prevent agglomeration and enhance the effectiveness of nanoparticles as antimicrobial agents. This property results in natural plant silver nanoparticles exhibiting superior stability and more potent antimicrobial effects [121]. The primary antimicrobial mechanism of zinc oxide nanoparticles (ZnO-NPs) involves the generation of ROS, which can target multiple sites, such as proteins, nucleic acids, lipids, and enzymes [125]. Compared to traditional ABHSGs, ZnO-NPs demonstrate enhanced antimicrobial activity and greater safety, along with high biocompatibility [118].

Although the systemic toxicity of nanoformulations as nanoscale materials has not been reported, some studies have shown that nanoformulations can easily interfere with biomolecules, cells, and organs [126]. Researchers are also concerned about the ability of nanoformulations to cross the blood–brain barrier via trans-synaptic transport, and accumulate in the brain [127]. Another issue with nanoformulations is that they pose a potential hazard to marine life if released into the environment, so future safety assessments for nanoformulations must be carried out to ensure they are safe for humans and the environment [128].

The utilization of nanoformulations in NABHSGs offers a promising avenue for developing hand hygiene products with improved antimicrobial efficacy, stability, and safety profiles. These advancements align with the growing demand for effective and safe hand hygiene solutions. Further research in this area can explore the optimization of nanoformulations, integration with natural active substances, and their potential applications in preventing the spread of infections and controlling disease transmission.

## 6. Future Research on HSGs

In the future, research in the field of novel HSGs should align with the increasing demands for safety and functionality among consumers [129]. The focus of such research may revolve around two main areas: Firstly, there is a need to screen natural components that possess low toxicity and demonstrate high antimicrobial performance. These components can be utilized as novel ingredients in HSGs [130]. Additionally, exploring the combination of multiple bioactive ingredients may further enhance the biological activities of HSGs. This approach allows for the development of multifunctional HSGs that not only sanitize hands effectively but also offer supplementary benefits [131]; secondly, optimizing the manufacturing processes of HSGs is crucial. This involves investigating gel materials that exhibit good biocompatibility, ensuring they are suitable for frequent use on the skin [132]. Rapid volatilization of the gel upon application can enhance the user experience and convenience. Moreover, exploring purely natural and edible components as gel materials holds the potential to increase the safety and acceptability of HSGs, addressing consumers’ growing concerns about environmental and health impacts [133].

By addressing these research directions, we can continuously improve and innovate the performance of HSGs, thereby enhancing their practicality among consumers. Novel HSGs will be better equipped to meet the public’s demand for safe, efficient, and eco-friendly hand cleaning solutions, contributing significantly to disease prevention and personal health maintenance [6]. These efforts will drive further advancements in the field of hand hygiene and provide us with more innovative and effective hand cleaning solutions.

## 7. Conclusions

Hand hygiene plays a key role in preventing and controlling infections, especially with the SARS-CoV-2 outbreak, which has seen a surge in demand for hand hygiene products. In this review, we provide a comprehensive overview of the various application scenarios, classifications, and challenges associated with HSGs. In addition, we highlight the emergence of novel ingredients with biological functions, which offer promising opportunities for advances in hand hygiene practice. By exploring multiple biological functions and ensuring ideal biosafety, future research could lead to the development of novel HSGs with excellent antimicrobial properties. Nevertheless, it is essential that we account for the potential risks of HSGs, such as the development of antimicrobial resistance and the impact of HSGs on the environment, while balancing their potential benefits. Better management and responsible hygiene practices should be promoted to ensure sustainable and effective hand hygiene solutions. In conclusion, this review provides valuable insights into hand hygiene and encourages further research and development of HSGs. As we continue to explore new possibilities and innovations in our human health goals, we remain committed to creating a safer, healthier, and more hygienic environment for all.

## Figures and Tables

**Table 1:** Different formulations of IHS and feature comparison.

The Formulations of IHS	Inactive Ingredients	Characteristics	Ref.
Liquid formulations	Humectant, fragrance, colorant.	Widely available, but with low viscosity and hard to dispense.	[17]
Spray formulations	Valve actuation, humectant, fragrance, colorant.	Higher flammability risk at room temperature.	[18]
Foam formulations	Foaming agent, humectant, fragrance, colorant.	Longer drying time, difficult to eliminate the feeling of dissimilarity and more expensive than gel.	[19,23]
Gel formulations	Emollients, thickeners, neutralizers, chelators, fragrances, and dyes or colorants.	With better antimicrobial action and fast drying time, formation of a protective layer on the application site.	[20,22]

## Data Availability

Not applicable.

## Abbreviations

IHSs	Instant hand sanitizers
HSGs	Hand sanitizer gels
ABHSGs	Alcohol-based hand sanitizer gels
NABHSGs	Non-alcohol-based hand sanitizer gels
QACs	Quaternary ammonium compounds
BZK	Benzalkonium chloride
EOs	Essential oils
EGCG	Epigallocatechin gallate
EC16	Epigallocatechin-3-gallate-palmitate
LUT	Luteolin
UVA	Ultraviolet A
UVB	Ultraviolet B
UV	Ultraviolet
ROS	Reactive oxygen species
OHE	*Opuntia humifusa* extract
HAS	Hyaluronic acid synthases
HA	Hyaluronic acid
HABP	Hyaluronic acid-binding protein
OAs	Organic acids
CA	Citric acid
MA	Mandelic acid
ZnAC	Zinc-amino clay
MDA	Malondialdehyde
ZnO-NPs	Zinc oxide nanoparticles
